# Role of Area-Level Access to Primary Care on the Geographic Variation of Cardiometabolic Risk Factor Distribution: A Multilevel Analysis of the Adult Residents in the Illawarra—Shoalhaven Region of NSW, Australia

**DOI:** 10.3390/ijerph17124297

**Published:** 2020-06-16

**Authors:** Renin Toms, Xiaoqi Feng, Darren J Mayne, Andrew Bonney

**Affiliations:** 1 School of Medicine, University of Wollongong, Wollongong NSW 2522, Australia; dmay8519@uni.sydney.edu.au (D.J.M.); abonney@uow.edu.au (A.B.); 2 Illawarra Health and Medical Research Institute, Wollongong NSW 2522, Australia; xiaoqi.feng@unsw.edu.au; 3 Population Wellbeing and Environment Research Lab (PowerLab), School of Health and Society, Faculty of Social Sciences, University of Wollongong, Wollongong NSW 2500, Australia; 4 School of Public Health and Community Medicine, University of New South Wales, Sydney NSW 2033, Australia; 5 Illawarra Shoalhaven Local Health District, Public Health Unit, Warrawong NSW 2502, Australia; 6 School of Public Health, The University of Sydney, Sydney NSW 2006, Australia

**Keywords:** geographic access, cardiometabolic risk factor, geographic variation, multilevel logistic regression, primary care access

## Abstract

*Background:* Access to primary care is important for the identification, control and management of cardiometabolic risk factors (CMRFs). This study investigated whether differences in geographic access to primary care explained area-level variation in CMRFs. *Methods:* Multilevel logistic regression models were used to derive the association between area-level access to primary care and seven discrete CMRFs after adjusting for individual and area-level co-variates. Two-step floating catchment area method was used to calculate the geographic access to primary care for the small areas within the study region. *Results:* Geographic access to primary care was inversely associated with low high density lipoprotein (OR 0.94, CI 0.91–0.96) and obesity (OR 0.91, CI 0.88–0.93), after adjusting for age, sex and area-level disadvantage. The intra-cluster correlation coefficient (ICCs) of all the fully adjusted models ranged between 0.4–1.8%, indicating low general contextual effects of the areas on CMRF distribution. The area-level variation in CMRFs explained by primary care access was ≤10.5%. *Conclusion:* The findings of the study support proportionate universal interventions for the prevention and control of CMRFs, rather than any area specific interventions based on their primary care access, as the contextual influence of areas on all the analysed CMRFs were found to be minimal. The findings also call for future research that includes other aspects of primary care access, such as road-network access, financial affordability and individual-level acceptance of the services in order to gain an overall picture of the area-level contributing role of primary care on CMRFs in the study region.

## 1. Introduction

Cardiometabolic risk factors (CMRFs) demonstrate significant variation in geographic distribution within countries globally [1,2,3,4,5,6,7,8,9,10]. Higher prevalence and clustering of CMRFs is often reported for socioeconomically disadvantaged areas [11,12,13,14,15,16,17,18,19,20]. Reachability or geographic access to primary care is essential for the individual-level identification and management of CMRFs, especially when considering their chronic nature after detection [21,22,23]. Therefore, access to primary care may be associated with the geographic variation of CMRFs [24].

Previous studies have reported that access to primary care can play a role in the control and management of certain CMRFs [21,25,26,27,28]. The dimensions of access to primary care can be fundamentally conceptualized into (1) physical access (2) affordability and (3) acceptability [29]. Research indicates that the physical access to primary care varies across areas, as the locations of primary care physicians and services tend to be positively correlated with population density [30,31]. There is also evidence that medical consultations were reported less likely to happen when physical access to health care services is lower [21]. In addition, access to adequate treatment facilities were reported to have an inverse association with certain CMRFs, such as hypertension [25,26], end stage renal disease [27] and diabetes mellitus [32]. However, these reports are based on individual CMRFs but consistent evidence across a range of CMRFs may provide a stronger evidence base for healthcare service commissioning across areas.

Evidence regarding the association of CMRFs with primary care access over and above area-level disadvantage may also inform area-level resource allocation of primary care services in disadvantaged areas [24,33]. Therefore, the aims of this study were to: (1) identify the area-level association of individual CMRFs with geographic access to primary care; (2) quantify the general contextual effect of areas on CMRFs; and (3) quantify the geographic variation in CMRFs explained by differences in area-level primary care access, within the Illawarra-Shoalhaven region of Australia.

## 2. Materials and Methods

A retrospective cross-sectional design and multilevel logistic regression models were adopted to meet the study objectives. The study was approved by the University of Wollongong and Illawarra and Shoalhaven Local Health District Health and Medical Human Research Ethics Committee (HREC protocol No: 2017/124).

**Figure 1:**
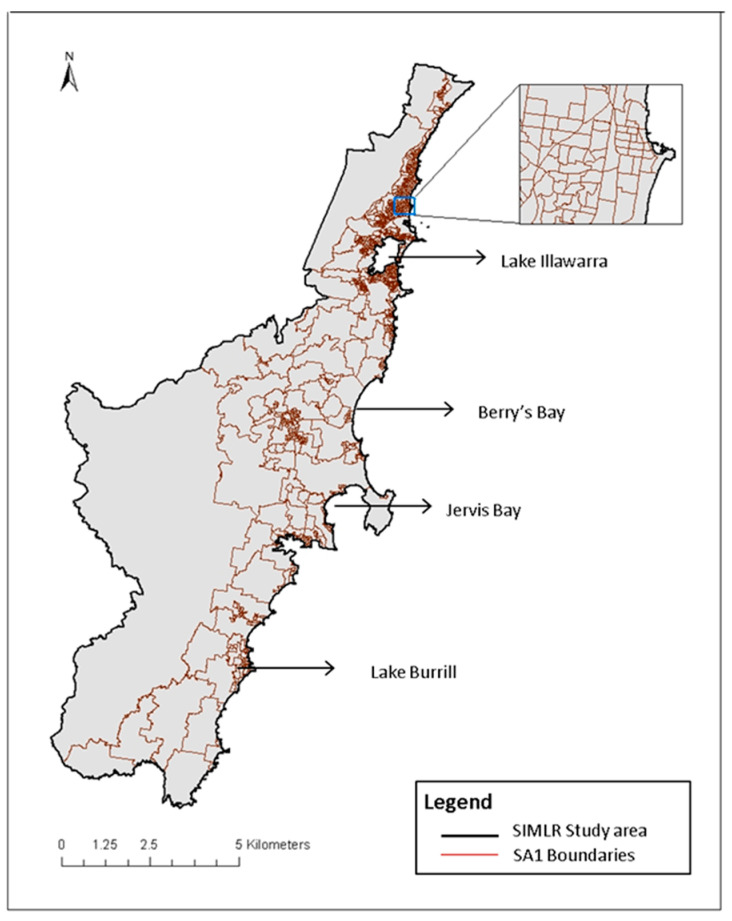
Map of the Illawarra-Shoalhaven region of NSW, Australia, showing SA1 areas and major landmarks.

The study focused on the Illawarra-Shoalhaven region of New South Wales (NSW), Australia. This area consists of multiple regional cities, smaller towns and rural areas, including the local government areas of Kiama, Shellharbour, Shoalhaven and Wollongong. The region covers a geographical area of 5615 square kilometres and had a population of 369,469 people at the 2011 Australian Census of Population and Housing [34,35]. The geographic unit of analysis used in this study was the Statistical Area 1 (SA1), which is the smallest statistical output unit of the 2011 Census and which has an average population of 400 people (range: 200 to 800) [36]. The study area encompassed 980 conterminous SA1s [37]. Figure 1 shows the study area showing SA1s and major landmarks of the region.

### 2.1. Data

The study used three different databases: (1) the CMRF pathology test data; (2) primary care provider data; and (3) the estimated resident populations from the 2011 Australian Census of Population and Housing. The CMRF test data were extracted from the Southern IML Research (SIMLR) Study database. The SIMLR Study database comprises de-identified and internally linked pathology results from a major pathology provider in the study region and provides near-census coverage of the study population [8]. The CMRF test data were extracted for multiple risk factors on the most recent test results, of non-pregnant adults aged 18 years and over, undergoing a laboratory test between 1 January 2012 and 31 December 2017.

The primary care provider data were manually extracted in 2016 from publicly available data sources, including Yellow Pages, White Pages, online general practitioner (GP) appointment booking services and Google search results. The 2011 Australian Census of Population and Housing data were accessed to extract the population denominator data of the study region at SA1 level [34].

### 2.2. Dependent Variable

Dichotomised results of the individual CMRF tests were the dependent variables in this study. The CMRF test results included: fasting blood sugar level (FBSL); glycated haemoglobin (HbA1c); total cholesterol (TC); high density lipoprotein (HDL); urinary albumin creatinine ratio (ACR); estimated glomerular filtration rate (eGFR); and objectively measured body mass index (BMI). The pathology service routinely collects BMI for each of the remaining CMRF tests and thus became available for analyses in this study [12]. However, it should be noted that the data do not include blood pressure readings. Although blood pressure is an important CMRF it is not routinely collected with any of the pathology test samples and thus not available for analyses in this study. During analyses, all the retrieved CMRF test results were dichotomised into higher risk and lower risk values based on established risk classification guideline values. Table 1 shows the CMRF definitions adopted in this study to dichotomies the test results.

### 2.3. Independent Variable

Primary care access index calculated for the small areas at SA1-level was the independent study variable. An access index score was calculated for each SA1 using a two-step floating catchment area (2SFCA) method, which balanced both supply and demand of primary care services in the study region.

The 2SFCA method was developed by Luo and Wang in 2003 to measure geographic accessibility of health care services [43]. The method has undergone several enhancements since its inception but essentially consists of two steps underpinned by a gravity model [43,44]. The first step computes a population-to-provider ratio for each primary care service location by aggregating the population size of the SA1s whose centroids (i.e., the geometric centers) are located within a defined spatial buffer distance [45]. The total number of general practitioners working in the primary care service locations within this buffer distance were the numerators for the provider-to-population ratio calculations.

Thus, step 1:

(1) Rj=Sj∑ipi

where *Sj* is the number of general practitioners at location *j*, pi is the number of adult residents in the SA1s (those SA1 geographic centroids are located within the spatial buffer distance of the primary care locations) and *Rj* is the population-to-provider ratio for service j [45].

In step 2, a population-to-provider ratio (access score) is computed for each geographic centroid of the SA1s by aggregating all primary care service population-to provider ratios of the primary care services that are located within the same spatial buffer distance [45].

Thus, step 2:

(2) $$A_{i}=\sum_{j}R_{j}$$

where *A_i_* is the access index for population location *i*.

The resulting access indices were retained as a continuous variable for the analyses. A higher score indicated better geographic access of the SA1s to primary care services.

A spatial radial buffer distance of 30 km was chosen to compute primary care access for SA1s in the study region. In the preliminary stage, sensitivity analyses were performed using 1 km, 16 km and 30 km spatial radial buffer distances. In step 1 2SFCA analyses, the 1 km distance covered only 545 (56%) SA1 centroids in the study region in relation to the primary care provider locations, whereas a 16 km radial buffer distance covered 973 (~99%) and a 30 km radial buffer distance covered 978 (~100%) SA1s’ geographic centroids. Therefore, a radial buffer distance of 30 km was chosen to determine the access which was observed to cover the mixed rural, semi-rural and urban distribution of the population in the study region well.

### 2.4. Covariates

The individual-level variables adjusted at SA1-level were: sex (male and female) and age group (18–29, 30–39, 40–49, 50–59, 60–69, 70–79 and 80+ years). The area-level covariate adjusted at SA1-level was the area-level socioeconomic disadvantage. The Index of Relative Socioeconomic Disadvantage (IRSD) score of the SA1s in the study region based on the 2011 Australian Bureau of Statistics conducted census of population and housing was used as the measurement variable for the area-level socioeconomic disadvantage of the SA1s [37]. The IRSD summarises a range of measures of relative socioeconomic disadvantage of people and/or households within SA1s and includes: level of income; education; employment; family structure; disability; housing; transportation; and internet connection [37]. A higher IRSD score indicated lower levels of disadvantage [37]. The Illawarra-Shoalhaven region has a diverse socio-economic profile, making this landscape useful for area-level population health studies [46].

### 2.5. Statistical Analyses

Multilevel logistic regression models were fitted to individual CMRF test data at the SA1 level. For each of the seven CMRFs analysed in this study, five nested models were fit that included fixed effects for access index after adjusting for sex, age and IRSD score; and random effect intercepts for SA1s. In the nested models, Model 1 (M1) was a null model of CMRF at SA1-level; Model 2 (M2) included the area-level study variable (access index) only; Model 3 (M3) included individual-level factors at SA1-level (age and sex) only; Model 4 (M4) included individual and area-level factors (age, sex and IRSD score) at SA1-level; and Model 5 (M5) included M4 variables plus access index. Thus, the final model (M5) estimated the effect of primary care access after adjusting for individual and area-level factors. Odds ratios (ORs) were derived from the exponentials of regression coefficients from fitted models. As the IRSD scores and access index of the SA1s were fitted as mean-centred continuous variables, ORs were expressed per standard deviation unit change in these variables. Statistical significance of the models was evaluated using likelihood ratio tests and a type I error rate of 0.05.

### 2.6. Model Comparison

Model fit was compared using the Akaike Information Criterion (AIC). The models were also evaluated for: area-level variance (τ^2^); proportional change in variance (PCV) in comparison with the null model; intra-cluster correlation coefficient (ICC) of the model; and the median odds ratios (MORs). The ICC and MOR of the models were used to index the between-area variability. A latent variable approach was used to derive the ICC of models [47]. The MOR translates the area-level variance into an easily interpretable OR and is assumed to be statistically independent of the test specific prevalence of the CMRFs [48]. The unique contribution of the primary care access of the SA1s to the area-level variance of CMRF was estimated through the reduction in PCV between M4 and M5.

### 2.7. Statistical Package

All mapping and geospatial measurements were performed using ArcGIS version 10.4.1 (ESRI Inc. Redlands, CA, USA) [49]. All statistical analyses were performed using R version 3.4.4. (R Foundation for Statistical Computing, Vienna, Austria) [50]. Multilevel models were fit using the glmer function in the lme4 package [51]; and likelihood ratio tests were calculated using the lrtest function in the lmtest package [52]. The glmer function fit the generalized linear mixed model, which incorporates both fixed-effects parameters and random effects in a linear predictor, via maximum likelihood [53,54].

## 3. Results

A total of 1,132,029 CMRF test results for 256,525 individual residents in the Illawarra-Shoalhaven region between 2012 and 2017 were extracted for analysis. The mean number of tests undertaken per person was 4.4 (SD = 1.8, range = 1–7). After excluding 1162 (1.0%) test results with incomplete details, a total of 1,130,894 tests were retained in the final data set. IRSD scores of the SA1s were the most frequent missing variable, as this was not available for some SA1s in the study region [55]. Available IRSD scores ranged between 446.7 and 1143.7 (mean = 976.7, SD = 98.6) for SA1s, with a higher score indicating lower area-level disadvantage. Table 2 details the individual-level CMRFs risk proportions of the final data set.

**Figure 2:**
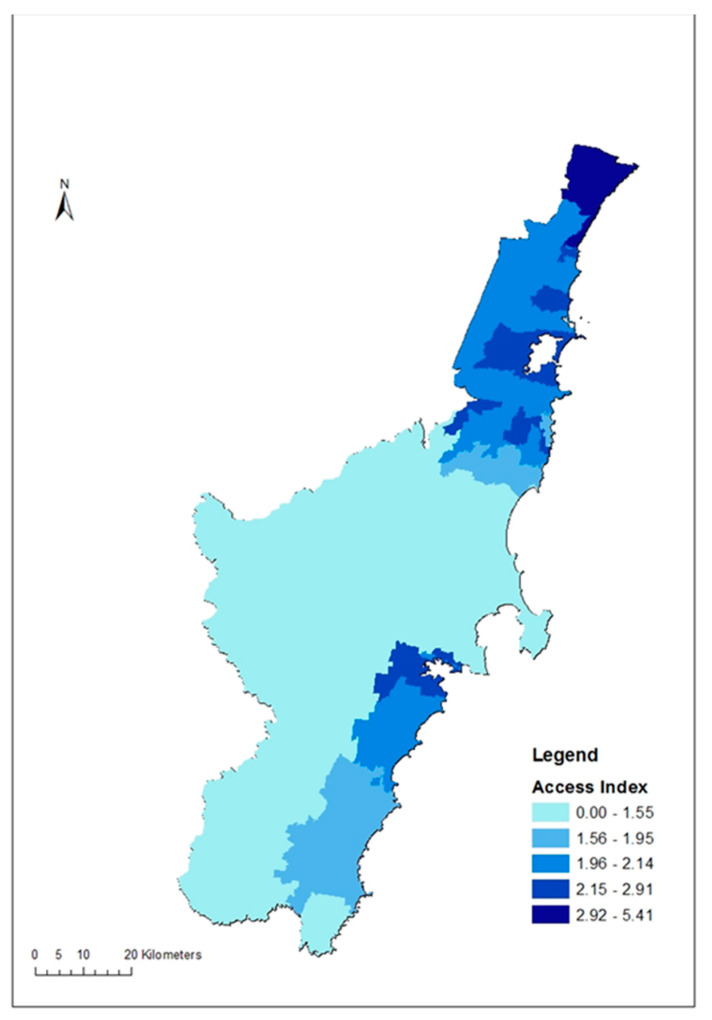
Geographic access to primary care services in the Illawarra-Shoalhaven region of the NSW, Australia.

For primary care access, a total of 165 primary care service locations with 611 general practitioners were identified in the study area in 2016. The primary care access index of the SA1s in the study region ranged between 0 and 5.41 general practitioners per 1000 people (mean = 2.1, SD = 0.77). Figure 2 illustrates the distribution of the primary care access index within the study region.

Multilevel logistic regression models for each CMRF are presented in Table 3, Table 4, Table 5, Table 6, Table 7, Table 8 and Table 9. The null models indicated significant geographic variation in the distribution of all CMRFs at the SA1 level. Model 2s showed inverse associations between access index and all CMRFs except TC, which displayed a positive association with the access index. Model 3s adjusted CMRF models for individual-level age and sex, which accounted for 1.5% (obesity) to 87.3% (eGFR) of unexplained variation in the null model. The general contextual effect of areas over and above their individual composition, such as age and sex, is obtained by a measure of clustering (i.e., ICCs) in the model 3s, which ranged between 0.6–3.4% in the CMRFs models presented. Model 4s demonstrated significant inverse associations between area-level socioeconomic disadvantage and all CMRFs except for TC after adjusting for individual-level factors. Total cholesterol again showed a positive association with area-level socioeconomic disadvantage. In the final models (M5s), the access index was found to be inversely associated with low HDL (HDL < 1 mmol/L) and obesity (BMI ≥ 30 kg/m^2^), after adjusting for individual and area-level factors. Including the access index in the final models did not attenuate associations between area-level disadvantage and CMRFs observed in M4s.

Model fit of the nested models of each CMRF were compared using the Akaike Information Criterion (AIC). The AIC estimated the out-of-sample prediction error rates of individual models and thus the relative quality of individual models for a given set of nested models [56]. Reductions in the AIC values were observed for all CMRFs models, except in TC and eGFR, from the null model (M1) to the final model (M5), indicating a better fit of the final models. The AIC for TC and eGFR models indicated M4 was the best fitting model for these CMRFs.

In the null models (M1s), low eGFR demonstrated the most area-level variance and high TC showed the least. The access only models (M2s) showed a reduction in the residual variance of all CMRFs from those of null models. In Model 3s, adjusting for age and sex initially increased the residual variance of FBSL (PCV = +1.9%), HbA1c (PCV = +3.0%), HDL (PCV = +15.3%) and BMI (PCV = +1.5%). In Model 4s, adjusting the CMRFs for individual-level age and sex and area-level disadvantage resulted in major reductions of variance from −33.1% (in TC) to −93.3% (in eGFR). In the final models (M5s), including access index in the models after adjusting for the covariates extended the reduction in variance in all CMRFs, except for TC and eGFR. Including the access index had been observed to increase the variance in the TC and eGFR final models, compared with the lower level model.

Similarly, in the unadjusted models, the MORs, which indicate the odds of having a higher risk CMRF test result for a person from the most, compared to the least, area-level disadvantage, were the highest among eGFR (τ^2^ = 0.189; ICC = 5.4%; MOR = 1.51) and the least among TC (τ^2^ = 0.025; ICC = 0.8%; MOR = 1.16). The ICCs of CMRFs in all the models were comparatively small (Table 4) in all the models, indicating minimal contextual effect of areas on any of the CMRFs. In the fully adjusted models, the ICCs further reduced and ranged between 0.4% and 1.8% in low eGFR and BMI respectively. Table 10 presents a summary and comparison of the model fit.

## 4. Discussion

This study aimed to inform area-specific interventions for the prevention and control of CMRFs, based on the primary care access status of the small areas within Illawarra-Sholhaven region of NSW, Australia. After adjusting for the covariates, we found that: a) greater access to primary care was associated with a reduction in the odds of low HDL and obesity but was not associated with high FBSL, high HbA1C, high TC, high ACR and low eGFR; b) the general contextual effect of areas on each of the CMRFs were minimal; and c) the geographic variation of CMRFs specifically explained by primary care access was small and did not demonstrate any attenuating effect on the contribution of area-level disadvantage on the variation of CMRFs in the study region. The results demonstrate that though the probability of low HDL and obesity decreases with increasing primary care access, the low general contextual effects of the areas on each of the CMRFs (i.e., low ICCs of Model 3s, ranges 0.6–3.4%) indicate minimal difference between the small areas after controlling for the study variables. Thus, the findings suggest that preventive interventions should not only be focused on areas with lower primary care access. Rather, interventions should be universal but proportional to the need and risk level of the people for the prevention and control of CMRFs. Primary care access was associated with all CRMFs in unadjusted models but only with low HDL and obesity in models fully adjusted for individual- and area-level covariates. These findings support the arguments of the possible role of confounders and reverse causality in ecological models [57], which question the previously established associations between primary care access and improved health [58,59]. The study suggests higher odds of being identified with low HDL and obesity with reduced access to primary care. In previous studies, when the relationship between health care service outcomes and travel time was modelled using multilevel logistic regression, it was found that GP consultations were less likely to happen when the travel time was longer, which is more common in rural areas [21]. The current study outcomes are consistent with those findings. However, it should also be noted that the current findings pertain only to the geographical/spatial accessibility of the primary health care services within 30 km distance of an SA1 centroid, rather than their road network access, actual usage and affordability.

The primary care access index, derived from the study region, ranged from 0 to 5.41 general practitioners per 1000 people (mean = 2.1, SD = 0.77). Multiple previous studies have reported inequalities in the geographic access to primary care services, using different enhanced versions of the 2SFCA method [45,60,61,62,63,64,65,66,67,68]. For example, the spatial accessibility index derived from rural Otago in New Zealand, using the travel time distance, ranged between 1 to 10, where a higher score indicated better access [62]. The accessibility index reported from Thimphu district in Bhutan ranged between 0 and 1, where 1 was the maximum access [69]. The spatial accessibility index of GP accessibility in England has been reported to range between 7.2 (South of England) and 13.3 (in London) [69]. The access map of the study region (Figure 2) clearly shows a polarisation of the higher access indices along the northern and southern ends of the study region, thus a visible inequality in their distribution. The WHO recommends universal access to primary care for all populations, where geographic access is one part of physical access to primary care [70].

Area-level disadvantage explained more geographic variation in CMRFs than area-level access to primary care. Inclusion of the access index in the final model did not demonstrate any reduction in the variance explained by area-level disadvantage on the geographic variation of CMRFs. This finding supports the importance of overall socioeconomic development of areas to reduce CMRF risk. Moreover, the ICC values of the final models were too small to suggest any meaningful area-level difference in the modelled CMRF variables. This would support the call for universal approaches for the prevention and control of CMRFs rather than any targeted area-level approaches, but with a proportional priority to disadvantaged populations in the study region [24,28,71].

This study has to be considered within its limitations. First, the cross-sectional nature of the study does not support causal inference. Second, the CMRF data used in this study are from people already utilising health care service in the study area, so care should be taken in generalising the results to the overall population. The SIMLR database does not include hospital or emergency service based tests. Therefore we believe that the database has a reasonable representation of community dwelling adults in the study region. However, it should be noted that the study sample includes only people who have accessed health care and pathology services, and the omission of those who have not accessed care may have biased our results. Given our population coverage this seem unlikely. Third, the study used a radial buffer distance of 30km for access calculations rather than travel time/distance because proprietary road network data were unavailable for this study. Thus, the patients’ actual experiences of seeking physical access in daily life need not exactly reflect the compound measure of access index adopted in this study. Even though the 30km buffer distance helped to include a maximum coverage of the population in relation to the geographic location of the primary care providers, this distance might have also influenced the discriminatory accuracy of the SA1s in the multilevel analyses. In addition, it should also be noted that the access index described in the study pertains only to the geographical reachability, but not to the affordability and acceptability of the available services. Forth, the study did not include blood pressure as a variable, although it is a major CMRF, due to non-availability of data. We were also unable to adjust for ethnicity for the same reason.

The main strength of this study is the use of a large population-derived database comprising a wide range of CMRFs. The research adds to the very few studies which consider multiple CMRF variables from the same region [18,19,20,72,73,74] and is unusual for its hierarchical analysis of the associations between a range of CMRFs and primary care access in a widely dispersed population.

Future research is required to investigate other area-level attributes contributing to the geographic variation of CMRFs in the study region. Our previous research has reported that area-level disadvantage contributes 14.7–57.8% of the geographic variation in CMRFs. The current study extended the previous findings by identifying the specific contribution of area-level primary care access, ranging between 0.0–10.5%. Further area-level analyses are required to identify other factors contributing to the geographic inequality of the CMRFs in the study region.

## 5. Conclusions

The findings of the study suggest that adults residing in areas that have a poor primary care access are more likely to be identified with low HDL and obesity. However, the specific contribution of area-level primary care access was small when compared to the contribution of area-level disadvantage. The finding supports the importance of overall socioeconomic development of areas to reduce CMRF risk, while supporting universal approaches for the prevention and control of CMRFs which are proportional to the need and disadvantage level of the individuals. Future research including other aspects of primary care access such as road-network access, financial affordability and acceptance of the services might help to provide an overall picture of the contributing role of primary care in the study region.

## Figures and Tables

**Table 1:** Definitions of CMRF (cardiometabolic risk factor) test results.

	Higher Risk CMRFs	Definition
1.	High FBSL	FBSL ≥ 7.0 mmol/L [38].
2.	High HbA1c	HbA1c > 7.5% [38].
3.	High TC	TC ≥ 5.5 mmol/L [39].
4.	Low HDL	HDL < 1 mmol/L [40].
5.	High ACR	ACR ≥ 30 mcg/L to mg/L [41].
6.	Low eGFR	eGFR < 60 mL/min/1.73 m^2^ [41].
7.	Obesity	BMI ≥ 30 kg/m^2^ [42].

CMRFs—cardiometabolic risk factors; FBSL—fasting blood sugar level; HbA1c—glycated haemoglobin; TC—total cholesterol; HDL—high density lipoprotein; ACR—albumin creatinine ratio; eGFR—estimated glomerular filtration rate; BMI—body mass index.

**Table 2:** Frequency and proportion of CMRFs risk classification with gender.

	Cardiometabolic Risk	Test *n*	Higher Risk *n* (%) *	Male *n* (%) *	Female *n* (%) *
1.	High FBSL	193,679	16,280 (8.4)	9289 (4.8)	6991 (3 .6)
2.	High HbA1c	73,885	7927 (10.7)	4448 (6.0)	3479 (4.7)
3.	High TC	194,816	63,422 (32.6)	26,139 (13.4)	37,283 (19.1)
4.	Low HDL	182,237	21,261 (11.7)	15,885 (8.7)	5376 (3.0)
5.	High ACR	50,790	2047 (4.0)	1266 (2.5)	781 (1.5)
6.	Low eGFR	244,166	27,241 (11.2)	12,456 (5.1)	14,785 (6.1)
7.	Obesity	192,455	64,832 (33.7)	29,613 (15.4)	35,319 (18.4)

* The denominators of the percentages are the total number of each CMRF tests.

**Table 3:** Multilevel logistic regression model summaries of high FBSL (FBSL ≥ 7.0 mmol/L).

Variables	Model 1		Model 2		Model 3		Model 4		Model 5	
Significance (LRT)	*p* < 0.0001		*p* < 0.0001		*p* < 0.0001		*p* < 0.0001		*p* < 0.0001	
	OR (95% CI)	*p* Value	OR (95% CI)	*p* Value	OR (95% CI)	*p* Value	OR (95% CI)	*p* Value	OR (95% CI)	*p* Value
High FBSL										
Intercept	0.09 (0.09–0.09)	*p* < 0.001	0.09 (0.09–0.09)	*p* < 0.001	0.01 (0.01–0.01)	*p* < 0.001	0.01 (0.01–0.01)	*p* < 0.001	0.01 (0.01–0.01)	*p* < 0.001
Access			0.89 (0.87–0.92)	*p* < 0.001						
Sex: Female					Reference					
Male					1.63 (1.58–1.69)	*p* < 0.001	1.63 (1.58–1.69)	*p* < 0.001	1.63 (1.58–1.69)	*p* < 0.001
Age: 18–29					Reference					
30–39					1.63 (1.40–1.90)	*p* < 0.001	1.65 (1.41–1.92)	*p* < 0.001	1.65 (1.41–1.92)	*p* < 0.001
40–49					3.53 (3.08–4.05)	*p* < 0.001	3.57 (3.11–4.10)	*p* < 0.001	3.57 (3.11–4.10)	*p* < 0.001
50–59					6.77 (5.93–7.72)	*p* < 0.001	6.81 (5.97–7.77)	*p* < 0.001	6.80 (5.97–7.75)	*p* < 0.001
60–69					11.07 (9.72–12.6)	*p* < 0.001	11.07 (9.7–12.6)	*p* < 0.001	11.05 (9.7–12.6)	*p* < 0.001
70–79					13.93 (12.2–15.9)	*p* < 0.001	13.8 (12.1–15.7)	*p* < 0.001	13.8 (12.1–15.7)	*p* < 0.001
80+					12.33 (10.8–14.1)	*p* < 0.001	12.1(10.6–13.9)	*p* < 0.001	12.1(10.6–13.8)	*p* < 0.001
IRSD							0.79 (0.77–0.80)	*p* < 0.001	0.79 (0.77–0.81)	*p* < 0.001
Access									0.98 (0.96–1.00)	0.111
AIC	111,022.8		110,962.3		103,066.2		102,652.6		102,652.0	
Variance	0.101		0.091		0.103		0.040		0.039	
PCV	-		−9.98%		+1.88%		−60.90%		−61.05%	
ICC (%)	3.0		2.7		3.0		1.2		1.2	
MOR	1.36		1.334		1.36		1.209		1.209	
Proportional variance explained by Access to primary care: −0.38%										

AIC—Akaike Information Criterion; FBSL—fasting blood sugar level; ICC—Intra-cluster correlation coefficient; IRSD—Index of Relative Socioeconomic Disadvantage; LRT—Likelihood ratio test; Model 1—null model at SA1 level; Model 2—M1 + Primary care access index of SA1s; Model 3—M1 + individual-level: age + sex; Model 4—Model 3 + Area level: Index of Relative Socioeconomic Disadvantage score of SA1s; Model 5—Model 4 + Primary care access index of SA1s; SA1—Statistical area-level 1; MOR—Median odds ratio; PCV—Proportional change in variance.

**Table 4:** Multilevel logistic regression model summaries of high HbA1c (HbA1c > 7.5%).

Variables	Model 1		Model 2		Model 3		Model 4		Model 5	
Significance (LRT)	*p* < 0.0001		*p* < 0.0001		*p* < 0.0001		*p* < 0.0001		*p* < 0.0001	
	OR (95% CI)	*p* Value	OR (95% CI)	*p* Value	OR (95% CI)	*p* Value	OR (95% CI)	*p* Value	OR (95% CI)	*p* Value
High HbA1c										
Intercept	0.12 (0.11–0.12)	*p* < 0.001	0.12 (0.11–0.12)	*p* < 0.001	0.07 (0.06–0.07)	*p* < 0.001	0.07 (0.06–0.08)	*p* < 0.001	0.07 (0.06–0.08)	*p* < 0.01
Access			0.95 (0.92–0.98)	*p* < 0.001						
Sex: Female					Reference					
Male					1.38 (1.3–1.45)	*p* < 0.001	1.39 (1.32–1.45)	*p* < 0.001	1.39 (1.32–1. 45)	*p* < 0.001
Age: 18–29					Reference					
30–39					0.81 (0.68–0.96)	*p* < 0.01	0.81 (0.68–0.96)	*p* < 0.01	0.81 (0.68–0. 97)	*p* < 0.01
40–49					1.24 (1.07–1.44)	*p* < 0.001	1.25 (1.08–1.45)	*p* < 0.001	1.26 (1.08–1. 46)	*p* < 0.001
50–59					1.56 (1.36–1.80)	*p* < 0.001	1.56 (1.36–1.80)	*p* < 0.001	1.57 (1.36–1. 81)	*p* < 0.001
60–69					1.64 (1.43–1.88)	*p* < 0.001	1.64 (1.43–1.88)	*p* < 0.001	1.64 (1.43–1. 89)	*p* < 0.001
70–79					1.64 (1.42–1.88)	*p* < 0.001	1.62 (1.41–1.86)	*p* < 0.001	1.63 (1.42–1. 87)	*p* < 0.001
80+					1.63 (1.41–1.88)	*p* < 0.001	1.62 (1.40–1.87)	*p* < 0.001	1.62 (1.41–1. 87)	*p* < 0.001
IRSD							0.79 (0.77–0.81)	*p* < 0.001	0.79 (0.77–0. 81)	*p* < 0.001
Access									1.00 (0.97–1.03)	0.750
AIC	50,114.5		50,105.9		49,690.2		49,438.2		49,440.0	
Variance	0.103		0.100		0.106		0.048		0.047	
PCV	-		−2.430%		+3.02%		−53.78%		−53.80%	
ICC (%)	3.0		3.0		3.1		1.4		1.4	
MOR	1.36		1.353		1.358		1.231		1.231	
Proportional variance explained by Access to primary care: –0.04%										

AIC—Akaike Information Criterion; HbA1c—glycated haemoglobin; ICC—Intra-cluster correlation coefficient; IRSD—Index of Relative Socioeconomic Disadvantage; LRT—Likelihood ratio test; Model 1—null model at SA1 level; Model 2—M1 + Primary care access index of SA1s; Model 3—M1 + individual-level: age + sex; Model 4—Model 3 + Area level: Index of Relative Socioeconomic Disadvantage score of SA1s; Model 5—Model 4 + Primary care access index of SA1s; SA1—Statistical area-level 1; MOR—Median odds ratio; PCV—Proportional change in variance.

**Table 5:** Multilevel logistic regression model summaries of high TC (TC ≥ 5.5 mmol/L).

Variables	Model 1		Model 2		Model 3		Model 4		Model 5	
Significance (LRT)	*p* < 0.0001		*p* < 0.0001		*p* < 0.0001		*p* < 0.0001		*p* < 0.0001	
	OR (95% CI)	*p* Value	OR (95% CI)	*p* Value	OR (95% CI)	*p* Value	OR (95% CI)	*p* Value	OR (95% CI)	*p* Value
high TC										
Intercept	0.42 (0.41–0.43)	*p* < 0.001	0.42 (0.41- 0.43)	*p* < 0.001	0.20 (0.19–0.21)	*p* < 0.001	0.20 (0.19–0.21)	*p* < 0.001	0.20 (0.19–0.21)	*p* < 0.01
Access			1.02 (1.00–1.03)	*p* < 0.01						
Sex: Female					Reference					
Male					0.69 (0.68–0.71)	*p* < 0.001	0.69 (0.68–0.71)	*p* < 0.001	0.69 (0.68–0.71)	*p* < 0.001
Age: 18–29					Reference					
30–39					2.02 (1.91–2.14)	*p* < 0.001	2.01 (1.90–2.13)	*p* < 0.01	2.01 (1.90–2.13)	*p* < 0.001
40–49					3.01 (2.86–3.17)	*p* < 0.001	3.00 (2.85–3.16)	*p* < 0.001	3.00 (2.85–3.16)	*p* < 0.001
50–59					4.08 (3.88–4.29)	*p* < 0.001	4.07 (3.87–4.28)	*p* < 0.001	4.07 (3.87–4.28)	*p* < 0.001
60–69					2.95 (2.80–3.10)	*p* < 0.001	2.95 (2.80–3.10)	*p* < 0.001	2.95 (2.80–3.10)	*p* < 0.001
70–79					1.60 (1.52–1.69)	*p* < 0.001	1.61 (1.52–1.69)	*p* < 0.001	1.61 (1.52–1.69)	*p* < 0.001
80+					1.13 (1.07–1.20)	*p* < 0.001	1.14 (1.07–1.21)	*p* < 0.001	1.14 (1.07–1.21)	*p* < 0.001
IRSD							1.06 (1.04–1.07)	*p* < 0.001	1.06 (1.04–1.07)	*p* < 0.001
Access									1.00 (0.98–1.01)	0.616
AIC	235,931.6		235,927.9		227,254.6		227,193.8		227,195.5	
Variance	0.0255		0.0250		0.020		0.01703		0.01705	
PCV	-		−1.69%		−21.76%		−33.11%		−33.07%	
ICC (%)	0.8		0.8		0.6		0.5		0.5	
MOR	1.16		1.163		1.14		1.133		1.133	
Proportional variance explained by Access to primary care: +0.12%										

AIC—Akaike Information Criterion; TC—total cholesterol; ICC—Intra-cluster correlation coefficient; IRSD—Index of Relative Socioeconomic Disadvantage; LRT—Likelihood ratio test; Model 1—null model at SA1 level; Model 2—M1 + Primary care access index of SA1s; Model 3—M1 + individual-level: age + sex; Model 4—Model 3 + Area level: Index of Relative Socioeconomic Disadvantage score of SA1s; Model 5—Model 4 + Primary care access index of SA1s; SA1—Statistical area-level 1; MOR—Median odds ratio; PCV—Proportional change in variance.

**Table 6:** Multilevel logistic regression model summaries of low HDL (<1 mmol/l).

Variables	Model 1		Model 2		Model 3		Model 4		Model 5	
Significance (LRT)	*p* < 0.0001		*p* < 0.0001		*p* < 0.0001		*p* < 0.0001		*p* < 0.0001	
	OR (95% CI)	*p* Value	OR (95% CI)	*p* Value	OR (95% CI)	*p* Value	OR (95% CI)	*p* Value	OR (95% CI)	*p* Value
low HDL										
Intercept	0.13 (0.13–0.13)	*p* < 0.001	0.13 (0.13–0.18)	*p* < 0.001	0.06 (0.06–0.07)	*p* < 0.001	0.06 (0.06–0.07)	*p* < 0.001	0.06 (0.06–0.07)	*p* < 0.001
Access			0.92 (0.90–0.94)	*p* < 0.001						
Sex: Female					Reference					
Male					3.98 (3.85–4.11)	*p* < 0.001	3.98 (3.85–4.11)	*p* < 0.001	3.98 (3.85–4.11)	*p* < 0.001
Age: 18–29					Reference					
30–39					1.11 (1.03–1.20)	*p* < 0.001	1.12 (1.04–1.21)	*p* < 0.001	1.12 (1.04–1.21)	*p* < 0.001
40–49					0.99 (0.92–1.05)	0.658	1.00 (0.93–1.07)	0.957	1.00 (0.93–1.07)	0.947
50–59					0.88 (0.82–0.94)	*p* < 0.001	0.89 (0.83–0.95)	*p* < 0.001	0.88 (0.83–0.95)	*p* < 0.001
60–69					0.82 (0.77–0.88)	*p* < 0.001	0.83 (0.77–0.88)	*p* < 0.001	0.82 (0.77–0.88)	*p* < 0.001
70–79					0.86 (0.80–0.92)	*p* < 0.001	0.85 (0.80–0.91)	*p* < 0.001	0.85 (0.79–0.91)	*p* < 0.001
80+					0.93 (0.86–1.00)	*p* < 0.010	0.92 (0.85–0.99)	*p* < 0.010	0.91 (0.85–0.99)	*p* < 0.010
IRSD							0.81 (0.80–0.82)	*p* < 0.001	0.82 (0.80–0.83)	*p* < 0.001
Access									0.95 (0.93–0.97)	*p* < 0.001
AIC	130,649.70		130,601.4		122,700.0		122,291.9		122,271.4	
Variance	0.07		0.064		0.081		0.031		0.029	
PCV	-		−9.48%		+15.25%		−55.90%		−59.05%	
ICC (%)	2.1		1.9		2.4		0.9		0.9	
MOR	1.289		1.273		1.313		1.183		1.183	
Proportional variance explained by Access to primary care: –6.61%									6.61%	

AIC—Akaike Information Criterion; HDL—high density lipoprotein; ICC—Intra-cluster correlation coefficient; IRSD—Index of Relative Socioeconomic Disadvantage; LRT—Likelihood ratio test; Model 1—null model at SA1 level; Model 2—M1 + Primary care access index of SA1s; Model 3—M1 + individual-level: age + sex; Model 4—Model 3 + Area level: Index of Relative Socioeconomic Disadvantage score of SA1s; Model 5—Model 4 + Primary care access index of SA1s; SA1—Statistical area-level 1; MOR—Median odds ratio; PCV—Proportional change in variance.

**Table 7:** Multilevel logistic regression model summaries of high ACR (≥30 mcg/L to mg/L).

Variables	Model 1		Model 2		Model 3		Model 4		Model 5	
Significance (LRT)	*p* < 0.0001		*p* < 0.0001		*p* < 0.0001		*p* < 0.0001		*p* < 0.0001	
	OR (95% CI)	*p* Value	OR (95% CI)	*p* Value	OR (95% CI)	*p* Value	OR (95% CI)	*p* Value	OR (95% CI)	*p* Value
High ACR										
Intercept	0.04 (0.04–0.04)	*p* < 0.001	0.04 (0.04–0.04)	*p* < 0.001	0.02 (0.02–0.03)	*p* < 0.001	0.02 (0.02–0.03)	*p* < 0.001	0.02 (0.02–0.03)	*p* < 0.001
Access			0.91 (0.86–0.96)	*p* < 0.001						
Sex: Female					Reference					
Male					1.75 (1.60–1.92)	*p* < 0.001	1.76 (1.60–1.93)	*p* < 0.001	1.75 (1.60–1.92)	*p* < 0.001
Age: 18–29					Reference					
30–39					1.00 (0.69–1.45)	0.985	1.01 (0.69–1.46)	0.978	1.00 (0.69–1.46)	0.982
40–49					0.69 (0.47–0.97)	*p* < 0.01	0.70 (0.50–1.00)	*p* < 0.01	0.70 (0.50–1.00)	*p* < 0.01
50–59					0.77 (0.56–1.05)	0.101	0.77 (0.56–1.07)	0.115	0.77 (0.56–1.06)	0.115
60–69					0.95 (0.70–1.30)	0.762	0.96 (0.71–1.31)	0.794	0.96 (0.70–1.30)	0.777
70–79					1.55 (1.15–2.10)	*p* < 0.001	1.55 (1.14–2.09)	*p* < 0.001	1.54 (1.14–2.08)	*p* < 0.001
80+					2.74 (2.02–3.71)	*p* < 0.001	2.71 (2.00–3.67)	*p* < 0.001	2.70 (1.99–3.66)	*p* < 0.001
IRSD							0.82 (0.78–0.85)	*p* < 0.001	0.82 (0.79–0.86)	*p* < 0.001
Access									0.97 (0.91–1.02)	0.206
AIC	17,130.0		17,119.9		16,585.2		16,510.8		16,511.2	
Variance	0.092		0.085		0.073		0.028		0.025	
PCV	-		−7.92%		−20.53%		−69.14%		−72.39%	
ICC (%)	2.7		2.5		2.2		0.9		0.8	
MOR	1.34		1.321		1.30		1.175		1.165	
Proportional variance explained by Access to primary care: −10.53%										

AIC—Akaike Information Criterion; ACR–albumin creatinine ratio; ICC—Intra-cluster correlation coefficient;IRSD—Index of Relative Socioeconomic Disadvantage; LRT—Likelihood ratio test; Model 1—null model at SA1 level; Model 2—M1 + Primary care access index of SA1s; Model 3—M1 + individual-level: age + sex; Model 4—Model 3 + Area level: Index of Relative Socioeconomic Disadvantage score of SA1s; Model 5—Model 4 + Primary care access index of SA1s; SA1—Statistical area-level 1; MOR—Median odds ratio; PCV—Proportional change in variance.

**Table 8:** Multilevel logistic regression model summaries of low eGFR (<60 mL/min/1.73 m^2^).

Variables	Model 1		Model 2		Model 3		Model 4		Model 5	
Significance (LRT)	*p* < 0.0001		*p* < 0.0001		*p* < 0.0001		*p* < 0.0001		*p* < 0.0001	
	OR (95% CI)	*p* Value	OR (95% CI)	*p* Value	OR (95% CI)	*p* Value	OR (95% CI)	*p* Value	OR (95% CI)	*p* Value
Low eGFR										
Intercept	0.11 (0.11–0.12)	*p* < 0.001	0.11 (0.11–0.12)	*p* < 0.001	0.00 (0.00–0.00)	*p* < 0.001	0.00 (0.00–0.00)	*p* < 0.001	0.00 (0.00–0.00)	*p* < 0.001
Access			0.89 (0.86–0.92)	*p* < 0.001						
Sex: Female					Reference					
Male					0.98 (0.95–1.01)	0.208	0.98 (0.95–1.01)	0.258	0.98 (0.95–1.01)	0.248
Age: 18–29					Reference					
30–39					1.66 (1.25–2.20)	*p* < 0.001	1.66 (1.24–2.23)	*p* < 0.001	1.65 (1.22–2.24)	*p* < 0.001
40–49					4.26 (3.35–5.41)	*p* < 0.001	4.27 (3.34–5.50)	*p* < 0.001	4.30 (3.32–5.58)	*p* < 0.001
50–59					12.26 (9.8–15.3)	*p* < 0.001	12.29 (9.73–15.52)	*p* < 0.001	12.28 (9.63–15.66)	*p* < 0.001
60–69					41.8 (33.6–51.8)	*p* < 0.001	41.84 (33.29–52.57)	*p* < 0.001	41.83 (32.97–53.06)	*p* < 0.001
70–79					150.7 (121.3–187.1)	*p* < 0.001	149.69 (119.3–187.9)	*p* < 0.001	149.6 (118.1–189.5)	*p* < 0.001
80+					509.3 (410.1–632.4)	*p* < 0.001	503.19 (400.9–631.6)	*p* < 0.001	503.0 (396.9–637.4)	*p* < 0.001
IRSD							0.90 (0.88–0.91)	*p* < 0.001	0.90 (0.88–0.91)	*p* < 0.001
Access									1.00 (0.98–1.02)	0.925
AIC	167,164.8		167,113.4		115,257.1		115,109.2		115,111.2	
Variance	0.189		0.176		0.024		0.013		0.013	
PCV	-		−6.53%		−87.26%		−93.31%		−93.26%	
ICC (%)	5.4		5.1		0.7		0.4		0.4	
MOR	1.51		1.492		1.16		1.113		1.113	
Proportional variance explained by Access to primary care: (+) 0.63%										

AIC—Akaike Information Criterion; eGFR—estimated glomerular filtration rate; ICC—Intra-cluster correlation coefficient; IRSD—Index of Relative Socioeconomic Disadvantage; LRT—Likelihood ratio test; Model 1—null model at SA1 level; Model 2—M1 + Primary care access index of SA1s; Model 3—M1 + individual-level: age + sex; Model 4—Model 3 + Area level: Index of Relative Socioeconomic Disadvantage score of SA1s; Model 5—Model 4 + Primary care access index of SA1s; SA1—Statistical area-level 1; MOR—Median odds ratio; PCV—Proportional change in variance.

**Table 9:** Multilevel logistic regression model summaries of obesity (BMI ≥ 30 kg/m^2^).

Variables	Model 1		Model 2		Model 3		Model 4		Model 5	
Significance (LRT)	*p* < 0.0001		*p* < 0.0001		*p* < 0.0001		*p* < 0.0001		*p* < 0.0001	
	OR (95% CI)	*p* Value	OR (95% CI)	*p* Value	OR (95% CI)	*p* Value	OR (95% CI)	*p* Value	OR (95% CI)	*p* Value
Obesity										
Intercept	0.51 (0.50–0.52)	*p* < 0.001	0.51 (0.50–0.52)	*p* < 0.001	0.25 (0.24–0.26)	*p* < 0.001	0.25 (0.24–0.25)	*p* < 0.001	0.25 (0.24–0.26)	*p* < 0.001
Access			0.88 (0.86–0.90)	*p* < 0.001						
Sex: Female					Reference					
Male					0.99 (0.97–1.01)	0.214	0.99 (0.97–1.01)	0.195	0.99 (0.97–1.01)	0.193
Age: 18–29					Reference					
30–39					1.63 (1.56–1.71)	*p* < 0.001	1.64 (1.57–1.71)	*p* < 0.001	1.64 (1.57–1.71)	*p* < 0.001
40–49					2.20 (2.11–2.29)	*p* < 0.001	2.21 (2.12–2.30)	*p* < 0.001	2.20 (2.12–2.30)	*p* < 0.001
50–59					2.44 (2.34–2.53)	*p* < 0.001	2.45 (2.35–2.54)	*p* < 0.001	2.44 (2.34–2.53)	*p* < 0.001
60–69					2.73 (2.63–2.84)	*p* < 0.001	2.74 (2.63–2.85)	*p* < 0.001	2.72 (2.62–2.83)	*p* < 0.001
70–79					2.44 (2.34–2.54)	*p* < 0.001	2.44 (2.34–2.54)	*p* < 0.001	2.42 (2.33–2.52)	*p* < 0.001
80+					1.46 (1.39–1.55)	*p* < 0.001	1.45 (1.38–1.54)	*p* < 0.001	1.45 (1.37–1.53)	*p* < 0.001
IRSD							0.81 (0.79–0.82)	*p* < 0.001	0.82 (0.80–0.83)	*p* < 0.001
Access									0.93 (0.91–0.95)	*p* < 0.001
AIC	242,793.2		242,686.2		239,122.6		238,731.8		238,680.6	
Variance	0.115		0.099		0.117		0.068		0.062	
PCV	-		−14.20%		+1.48%		−41.21%		−46.19%	
ICC (%)	3.4		2.9		3.4		2.0		1.8	
MOR	1.38		1.350		1.39		1.282		1.268	
Proportional variance explained by Access to primary care: –8.47%										

AIC—Akaike Information Criterion; BMI—body mass index; ICC—Intra-cluster correlation coefficient; IRSD—Index of Relative Socioeconomic Disadvantage; LRT—Likelihood ratio test; Model 1—null model at SA1 level; Model 2—M1 + Primary care access index of SA1s; Model 3—M1 + individual-level: age + sex; Model 4—Model 3 + Area level: Index of Relative Socioeconomic Disadvantage score of SA1s; Model 5—Model 4 + Primary care access index of SA1s; SA1—Statistical area-level 1; MOR—Median odds ratio; PCV—Proportional change in variance.

**Table 10:** Summary of model fit values and comparison of the models.

		FBSL	HbA1c	TC	HDL	ACR	eGFR	Obesity
**Model 1**	Null Model							
	AIC	111,022.8	50,114.5	235,931.6	130,649.7	17,130.0	167,164.8	242,793.2
	$\tau^{2}$	0.101	0.103	0.025	0.071	0.092	0.189	0.115
	ICC (%)	3.0	3.0	0.8	2.1	2.7	5.4	3.4
	MOR	1.36	1.36	1.16	1.29	1.34	1.51	1.38
**Model 2**	Access Model							
	AIC	110,962.3	50,105.9	235,927.9	130,601.4	17,119.9	167,113.4	242,686.2
	$\tau^{2}$	0.091	0.100	0.025	0.064	0.085	0.176	0.099
	ICC (%)	2.7	3.0	0.8	1.9	2.5	5.1	2.9
	MOR	1.334	1.353	1.163	1.273	1.321	1.492	1.350
	PCV	−9.98%	−2.430%	−1.69%	−9.48%	−7.92%	−6.53%	−14.20%
**Model 3**	Sex + Age Adjusted Model							
	AIC	103,066.2	49,690.2	227,254.6	122,700.0	16,585.2	115,257.1	239,122.6
	$\tau^{2}$	0.103	0.106	0.020	0.081	0.073	0.024	0.117
	ICC (%)	3.0	3.1	0.6	2.4	2.2	0.7	3.4
	MOR	1.36	1.358	1.14	1.31	1.30	1.16	1.39
	PCV	+ 1.88%	+ 3.02%	−21.76%	+15.25%	−20.53%	−87.26%	+1.48%
**Model 4**	Sex + Age + IRSD Adjusted Model							
	AIC	102,652.6	49,438.2	227,193.8	122,291.9	16,510.8	115,109.2	238,731.8
	$\tau^{2}$	0.040	0.048	0.017	0.031	0.028	0.013	0.068
	ICC (%)	1.2	1.4	0.5	0.9	0.9	0.4	2.0
	MOR	1.209	1.231	1.133	1.183	1.175	1.113	1.282
	PCV	−60.90%	−53.78%	−33.11%	−55.90%	−69.14%	−93.31%	−41.21%
**Model 5**	Sex + Age + IRSD Adjusted and Access included Model							
	AIC	102,652.0	49,440.0	227,195.5	122,271.4	16,511.2	115,111.2	238,680.6
	$\tau^{2}$	0.039	0.047	0.017	0.029	0.025	0.013	0.062
	ICC (%)	1.2	1.4	0.5	0.9	0.8	0.4	1.8
	MOR	1.209	1.231	1.133	1.183	1.165	1.113	1.268
	PCV	−61.05%	−53.80%	−33.07%	−59.05%	−72.39%	−93.26%	−46.19%

AIC—Akaike Information Criterion; τ^2^—residual variance; IC—intra-cluster correlation coefficients; MOR—median odds ratio; PCV—proportional change in variance; FBSL—fasting blood sugar level; HbA1c—glycated haemoglobin; TC—total cholesterol; HDL—high density lipoprotein; ACR—albumin creatinine ratio; eGFR—estimated glomerular filtration rate.

## References

[1] Alkerwi A., Bahi I.E., Stranges S., Beissel J., Delagardelle C., Noppe S., Kandala N.-B. (2017). Geographic variations in cardiometabolic risk factors in Luxembourg. Int. J. Environ. Res. Public Health.

[2] Astell-Burt T., Feng X., Kolt G.S., McLean M., Maberly G. (2014). Understanding geographical inequities in diabetes: Multilevel evidence from 114,755 adults in Sydney, Australia. Diabetes Res. Clin. Pract..

[3] Barker L.E., Kirtland K.A., Gregg E.W., Geiss L.S., Thompson T.J. (2011). Geographic distribution of diagnosed diabetes in the US: A diabetes belt. Am. J. Prev. Med..

[4] Congdon P. (2006). Estimating diabetes prevalence by small area in England. J. Public Health.

[5] Lawlor D., Bedford C., Taylor M., Ebrahim S. (2003). Geographical variation in cardiovascular disease, risk factors, and their control in older women: British Women’s Heart and Health Study. J. Epidemiol. Community Health.

[6] Paquet C., Chaix B., Howard N.J., Coffee N.T., Adams R.J., Taylor A.W., Thomas F., Daniel M. (2016). Geographic clustering of cardiometabolic risk factors in metropolitan centres in France and Australia. Int. J. Environ. Res. Public Health.

[7] Toms R., Bonney A., Mayne D.J., Feng X., Walsan R. (2019). Geographic and area-level socioeconomic variation in cardiometabolic risk factor distribution: A systematic review of the literature. Int. J. Health Geogr..

[8] Toms R., Mayne D.J., Feng X., Bonney A. (2019). Geographic variation in cardiometabolic risk distribution: A cross-sectional study of 256,525 adult residents in the Illawarra-Shoalhaven region of the NSW, Australia. PLoS ONE.

[9] Valdés S., García-Torres F., Maldonado-Araque C., Goday A., Calle-Pascual A., Soriguer F., Castaño L., Catalá M., Gomis R., Rojo-Martínez G. (2014). Prevalence of obesity, diabetes and other cardiovascular risk factors in Andalusia (southern Spain). Comparison with national prevalence data. The Di@bet. es study. Rev. Española Cardiol..

[10] Zhou M., Astell-Burt T., Bi Y., Feng X., Jiang Y., Li Y., Page A., Wang L., Xu Y., Wang L. (2015). Geographical variation in diabetes prevalence and detection in China: Multilevel spatial analysis of 98,058 adults. Diabetes Care.

[11] Andersen A., Carson C., Watt H., Lawlor D., Avlund K., Ebrahim S. (2008). Life-course socio-economic position, area deprivation and Type 2 diabetes: Findings from the British Women’s Heart and Health Study. Diabet. Med..

[12] Bonney A., Mayne D.J., Jones B.D., Bott L., Andersen S.E., Caputi P., Weston K.M., Iverson D.C. (2015). Area-level socioeconomic gradients in overweight and obesity in a community-derived cohort of health service users—A cross-sectional study. PLoS ONE.

[13] Cubbin C., Sundquist K., Ahlén H., Johansson S.E., Winkleby M.A., Sundquist J. (2006). Neighborhood deprivation and cardiovascular disease risk factors: Protective and harmful effects. Scand. J. Public Health.

[14] Dragano N., Bobak M., Wege N., Peasey A., Verde P.E., Kubinova R., Weyers S., Moebus S., Möhlenkamp S., Stang A. (2007). Neighbourhood socioeconomic status and cardiovascular risk factors: A multilevel analysis of nine cities in the Czech Republic and Germany. BMC Public Health.

[15] Lawlor D.A., Davey Smith G., Patel R., Ebrahim S. (2005). Life-course socioeconomic position, area deprivation, and coronary heart disease: Findings from the British Women’s Heart and Health Study. Am. J. Public Health.

[16] Maier W., Scheidt-Nave C., Holle R., Kroll L.E., Lampert T., Du Y., Heidemann C., Mielck A. (2014). Area level deprivation is an independent determinant of prevalent type 2 diabetes and obesity at the national level in Germany. Results from the National Telephone Health Interview Surveys ‘German Health Update’GEDA 2009 and 2010. PLoS ONE.

[17] Mujahid M.S., Diez Roux A.V., Borrell L.N., Nieto F.J. (2005). Cross-sectional and longitudinal associations of BMI with socioeconomic characteristics. Obes. Res..

[18] Naimi A.I., Paquet C., Gauvin L., Daniel M. (2009). Associations between area-level unemployment, body mass index, and risk factors for cardiovascular disease in an urban area. Int. J. Environ. Res. Public Health.

[19] Roux A.V.D., Jacobs D.R., Kiefe C.I. (2002). Neighborhood characteristics and components of the insulin resistance syndrome in young adults: The coronary artery risk development in young adults (CARDIA) study. Diabetes Care.

[20] Unger E., Diez-Roux A.V., Lloyd-Jones D.M., Mujahid M.S., Nettleton J.A., Bertoni A., Badon S.E., Ning H., Allen N.B. (2014). Association of neighborhood characteristics with cardiovascular health in the multi-ethnic study of atherosclerosis. Circulation.

[21] Hiscock R., Pearce J., Blakely T., Witten K. (2008). Is neighborhood access to health care provision associated with individual-level utilization and satisfaction?. Health Serv. Res..

[22] Schmidt M.I., Duncan B.B., e Silva G.A., Menezes A.M., Monteiro C.A., Barreto S.M., Chor D., Menezes P.R. (2011). Chronic non-communicable diseases in Brazil: Burden and current challenges. Lancet.

[23] Weinberger M., Oddone E.Z., Henderson W.G. (1996). Does increased access to primary care reduce hospital readmissions?. N. Engl. J. Med..

[24] Kirby J.B., Kaneda T. (2005). Neighborhood socioeconomic disadvantage and access to health care. J. Health Soc. Behav..

[25] Atallah A., Inamo J., Larabi L., Chatellier G., Rozet J., Machuron C., De Gaudemaris R., Lang T. (2007). Reducing the burden of arterial hypertension: What can be expected from an improved access to health care? Results from a study in 2420 unemployed subjects in the Caribbean. J. Hum. Hypertens.

[26] Kotchen J.M., Shakoor-Abdullah B., Walker W.E., Chelius T.H., Hoffmann R.G., Kotchen T.A. (1998). Hypertension control and access to medical care in the inner city. Am. J. Public Health.

[27] Occelli F., Deram A., Génin M., Noël C., Cuny D., Glowacki F. (2014). Mapping end-stage renal disease (ESRD): Spatial variations on small area level in northern France, and association with deprivation. PLoS ONE.

[28] Walsh M.G., Zgibor J., Songer T., Borch-Johnsen K., Orchard T.J., Investigators A.D. (2005). The socioeconomic correlates of global complication prevalence in type 1 diabetes (T1D): A multinational comparison. Diabetes Res. Clin. Pract..

[29] World Health Organization Gender, Equity and Human Rights: Accessibility. https://www.who.int/gender-equity-rights/understanding/accessibility-definition/en/.

[30] Angier H., Likumahuwa S., Finnegan S., Vakarcs T., Nelson C., Bazemore A., Carrozza M., DeVoe J.E. (2014). Using geographic information systems (GIS) to identify communities in need of health insurance outreach: An OCHIN practice-based research network (PBRN) report. J. Am. Board Fam. Med..

[31] Larkins S., Gupta T.S., Evans R., Murray R., Preston R. (2011). Addressing inequities in access to primary health care: Lessons for the training of health care professionals from a regional medical school. Aust. J. Prim. Health.

[32] Harris M.I. (2001). Racial and ethnic differences in health care access and health outcomes for adults with type 2 diabetes. Diabetes Care.

[33] Pattenden S., Casson K., Cook S., Dolk H. (2011). Geographical variation in infant mortality, stillbirth and low birth weight in Northern Ireland, 1992–2002. J. Epidemiol. Community Health.

[34] Australian Bureau of Statistics Census Data. https://www.abs.gov.au/websitedbs/censushome.nsf/home/historicaldata2011?opendocument&navpos=280.

[35] Australian Bureau of Statistics (2011). Australian Statistical Geography Standard (ASGS): Census Dictionary. http://www.abs.gov.au/ausstats/abs@.nsf/Lookup/2901.0Chapter23102011.

[36] Australian Bureau of Statistics (ASGS) Australian Statistical Geography Standard (ASGS): Volume 1—Main Structure: STATISTICAL AREA LEVEL 1 (SA1). https://www.abs.gov.au/websitedbs/D3310114.nsf/home/Australian+Statistical+Geography+Standard+.

[37] Australian Bureau of Statistics Main Features—IRSD. https://www.abs.gov.au/ausstats/abs@.nsf/Lookup/2033.0.55.001main+features100052011.

[38] The Royal Australian College of General Practitioners & Diabetes Australia General Practice Management of Type 2 Diabetes 2016–2018. https://static.diabetesaustralia.com.au/s/fileassets/diabetes-australia/5d3298b2-abf3-487e-9d5e-0558566fc242.pdf.

[39] Australian Bureau of Statistics Australian Health Survey: Biomedical Results for Chronic Diseases, 2011-12. https://www.abs.gov.au/AUSSTATS/abs@.nsf/DetailsPage/4364.0.55.0052011-12.

[40] National Heart Foundation of Australia Lipid Management Profile for Health Professionals. https://www.heartfoundation.org.au/for-professionals/clinical-information/lipid-management.

[41] National Kidney Foundation (USA) Albumin Creatinine Ratio (ACR). https://www.kidney.org/kidneydisease/siemens_hcp_acr.

[42] World Health Organization (2000). Obesity: Preventing and Managing the Global Epidemic: Technical Report Series.

[43] Luo W., Wang F. (2003). Measures of spatial accessibility to health care in a GIS environment: Synthesis and a case study in the Chicago region. Environ. Plan. B.

[44] Li Y., Vo A., Randhawa M., Fick G. (2017). Designing utilization-based spatial healthcare accessibility decision support systems: A case of a regional health plan. Decis. Support Syst..

[45] McGrail M.R. (2012). Spatial accessibility of primary health care utilising the two step floating catchment area method: An assessment of recent improvements. Int. J. Health Geogr..

[46] Ghosh A., Charlton K.E., Girdo L., Batterham M. (2014). Using data from patient interactions in primary care for population level chronic disease surveillance: The Sentinel Practices Data Sourcing (SPDS) project. BMC Public Health.

[47] Goldstein H., Browne W., Rasbash J. (2002). Partitioning variation in multilevel models. Underst. Stat..

[48] Szmaragd C., Leckie G. (2011). Module 6: Regression Models for Binary Responses R Practical.

[49] Environmental Systems Research Institute (ESRI) ArcGIS 10.4.1.

[50] R Core Team R: A Language and Environment for Statistical Computing.

[51] Bates D., Mächler M., Bolker B., Walker S. (2014). Fitting linear mixed-effects models using lme4. arXiv.

[52] Zeileis A., Hothorn T. Diagnostic Checking in Regression Relationships. http://CRAN.R-project.org/doc/Rnews/.

[53] Bolker B. R Documention: Fitting Generalized Linear Mixed-Effects Models. https://www.rdocumentation.org/packages/lme4/versions/1.1-23/topics/glmer.

[54] Liu Q., Pierce D.A. (1994). A note on Gauss—Hermite quadrature. Biometrika.

[55] Australian Bureau of Statistics Technical Paper: Socio-Economic Indexes for Areas (SEIFA). https://www.ausstats.abs.gov.au/Ausstats/subscriber.nsf/0/22CEDA8038AF7A0DCA257B3B00116E34/$File/2033.0.55.001%20seifa%202011%20technical%20paper.pdf.

[56] Aho K., Derryberry D., Peterson T. (2014). Model selection for ecologists: The worldviews of AIC and BIC. Ecology.

[57] Gulliford M.C. (2002). Availability of primary care doctors and population health in England: Is there an association?. J. Public Health.

[58] Shi L., Starfield B. (2001). The effect of primary care physician supply and income inequality on mortality among blacks and whites in US metropolitan areas. Am. J. Public Health.

[59] Shi L., Starfield B., Politzer R., Regan J. (2002). Primary care, self-rated health, and reductions in social disparities in health. Health Serv. Res..

[60] Bagheri N., Holt A., Benwell G.L. (2009). Using geographically weighted regression to validate approaches for modelling accessibility to primary health care. Appl. Spat. Anal. Policy.

[61] Bissonnette L., Wilson K., Bell S., Shah T.I. (2012). Neighbourhoods and potential access to health care: The role of spatial and aspatial factors. Health Place.

[62] Gruen R.L., Weeramanthri T.S., Knight S.E., Bailie R.S. (2006). Specialist outreach clinics in primary care and rural hospital settings (Cochrane Review). Community Eye Health..

[63] Kanuganti S., Sarkar A.K., Singh A.P., Arkatkar S.S. (2015). Quantification of accessibility to health facilities in rural areas. Case Stud. Transp. Policy.

[64] Mobley L.R., Root E., Anselin L., Lozano-Gracia N., Koschinsky J. (2006). Spatial analysis of elderly access to primary care services. Int. J. Health Geogr..

[65] Munoz U.H., Källestål C. (2012). Geographical accessibility and spatial coverage modeling of the primary health care network in the Western Province of Rwanda. Int. J. Health Geogr..

[66] Shah T.I., Milosavljevic S., Bath B. (2017). Measuring geographical accessibility to rural and remote health care services: Challenges and considerations. Spat. Spatio Temporal Epidemiol..

[67] Wang F., Luo W. (2005). Assessing spatial and nonspatial factors for healthcare access: Towards an integrated approach to defining health professional shortage areas. Health Place.

[68] Wang X., Yang H., Duan Z., Pan J. (2018). Spatial accessibility of primary health care in China: A case study in Sichuan Province. Soc. Sci Med..

[69] Bauer J., Müller R., Brüggmann D., Groneberg D.A. (2018). Spatial accessibility of primary care in England: A cross-sectional study using a floating catchment area method. Health Serv. Res..

[70] Evans D.B., Hsu J., Boerma T. (2013). Universal Health Coverage and Universal Access.

[71] Rose G. (1985). Sick individuals and sick populations. Int. J. Epidemiol..

[72] Clark C.R., Ommerborn M.J., Hickson D.A., Grooms K.N., Sims M., Taylor H.A., Albert M.A. (2013). Neighborhood disadvantage, neighborhood safety and cardiometabolic risk factors in African Americans: Biosocial associations in the Jackson Heart study. PLoS ONE.

[73] Gabert R., Thomson B., Gakidou E., Roth G. (2016). Identifying high-risk neighborhoods using electronic medical records: A population-based approach for targeting diabetes prevention and treatment interventions. PLoS ONE.

[74] Keita A.D., Judd S.E., Howard V.J., Carson A.P., Ard J.D., Fernandez J.R. (2014). Associations of neighborhood area level deprivation with the metabolic syndrome and inflammation among middle-and older-age adults. BMC Public Health.

